# Ectotherms in Variable Thermal Landscapes: A Physiological Evaluation of the Invasive Potential of Fruit Flies Species

**DOI:** 10.3389/fphys.2016.00302

**Published:** 2016-07-19

**Authors:** Francisca Boher, Nicole Trefault, Sergio A. Estay, Francisco Bozinovic

**Affiliations:** ^1^Departamento de Ecología, Facultad de Ciencias Biológicas, Pontificia Universidad Católica de ChileSantiago, Chile; ^2^Center of Applied Ecology and Sustainability, Pontificia Universidad Católica de ChileSantiago, Chile; ^3^Centro de Genómica y Bioinformática and Instituto de Biotecnología, Facultad de Ciencias, Universidad MayorSantiago, Chile; ^4^Facultad de Ciencias, Instituto de Ciencias Ambientales y Evolutivas, Universidad Austral de ChileValdivia, Chile

**Keywords:** global change, environmental variability, physiological acclimation, heat shock proteins

## Abstract

Climate change and biological invasions pose one of the greatest threats to biodiversity. Most analyses of the potential biological impacts have focused on changes in mean temperature, but changes in thermal variance may also impact native and invasive organisms, although differentially. We assessed the combined effects of the mean and the variance of temperature on the expression of heat shock protein (*hsp90*) in adults of the invasive fruit fly *Drosophila melanogaster* and the native *Drosophila gaucha* in Mediterranean habitats of central Chile. We observed that, under these experimental conditions, *hsp90* mRNA expression was higher in the invasive species but absent in the native one. Apparently, the biogeographic origin and niche conservatisms are playing a role in the heat shock response of these species under different putative scenarios of climate change. We suggest that in order to develop more realistic predictions about the biological impact of climate change and biological invasions, one must consider the interactions between the mean and variance of climatic variables, as well as the evolutionary original conditions of the native and invasive species.

## Introduction

The question about what makes an exotic organism a successful invader has been central in the fields of applied ecology and environmental protection the last decades (Kolar and Lodge, 2001; Seastedt, 2009). Current research points out to a mix between individual and community or ecosystem features. In the community approach, focus has been on the differential characteristics of susceptible and resistant communities (Hector et al., 2001), like the hypothesis of diversity—invasiveness. At individual level the search of traits that predict the invasive potential of a species spans from reproductive potential and foraging habits to environmental tolerances (Sol et al., 2012; Bates et al., 2013; Capellini et al., 2015). It is in the later where thermal physiology provides the conceptual framework needed to explain the success—failure pattern of exotic species over the thermal landscape.

How it has been previously described (Helmuth et al., 2010; Bozinovic et al., 2011a,b; Estay et al., 2014), insights about the suitability of a thermal landscape for a given species should make reference not only to average values, but also to the intrinsic variability of perceived temperatures (Bozinovic et al., 2016a,b). This is a key point at predicting future changes due to climate change, where theoretical (Katz et al., 2005) and empirical approaches (Easterling et al., 2000) indicate that global warming impacts not only the mean temperatures, but also the magnitude of dial and seasonal variation in temperature (Vazquez et al., 2016).

Ectotherms are particularly susceptible to temperature variation, as their body temperature is determined to a large extent by environmental conditions (Hoffmann et al., 2003; Karl et al., 2009). The ability to cope with extremes rather than different mean temperatures is probably of much greater importance for species survival and thermal adaptation (Anderson et al., 2003). The most studied physiological mechanism to cope with extreme temperatures is through the expression of stress-inducible heat-shock proteins (HSPs). These, chaperone proteins minimize the problems that arise when other proteins are in a non-native conformation (Feder, 1999; Feder and Hofmann, 1999). Most HSPs participate in protein folding and unfolding, and they are essential in cellular responses to a variety of damaging conditions (Parsell and Lindquist, 1994). Most of our knowledge about shifts in gene expression in response to changes in temperature comes from studies of the heat- and cold-shock responses (Feder and Hofmann, 1999; Johnson et al., 2009; Zhang and Denlinger, 2010). However, these studies typically focus on a narrow range of high or low temperatures that are severe and induce a strong cellular stress response. Very few studies have addressed changes in gene expression associated with routine daily or seasonal temperature regimes experienced by organisms (Podrabsky and Somero, 2004).

A simple hypothesis relating invasiveness and HSPs indicates that HSP expression is high in invasive species (Kelley, 2014); however, a new question arise: which is the base line to compare expression levels?. An elegant solution is compare expression levels between close related non-invasive and invasive species. Some evidence pointed out that invasive species are more eurythermal than natives, i.e., have the ability to maintain physiological function over a wide range of temperatures. Unfortunately, the few studies that compared temperature tolerances between invasive and native non-invasive species have shown conflicting results (see Kelley, 2014 meta-analysis). This lack of agreement in the results could be a consequence of the importance of the evolutionary history on the current status of traits linked to thermal tolerance. In this sense, the biogeographic origin of each species is a key component in this kind of comparative analysis.

Here, we experimentally test the effects of potential scenarios of climate warming given by changes in mean temperature and thermal variance on the heat shock protein response (*hsp90* mRNA) of an invasive and a native non-invasive species in central Chile. Specifically we address the following questions: (1) Does the expression of transcripts encoding for hsp90 vary across mean temperature and thermal variance treatments? (2) Does the expression of transcripts encoding for hsp90 vary between native and invasive species? and (3) How does putative climate change interact with the biogeographic origin of native and invasive species in the expression of hsp90 mRNA?

## Materials and methods

### Model species

Our study use two species of *Drosophila* as a model for answering our questions: *D. melanogaster* and *Drosophila gaucha*. The former is an invasive species with a tropical origin whose range expansion may be associated with human activities (Keller, 2007). According to the entomological literature, *D. melanogaster* was absent from Chile until 1888 (Blanchard, 1851; Reed, 1888), and the first records from Chile can be found in the work of Sturtevant (1921). On the other hand, *D. gaucha* is a native species that exhibit a comparatively smaller geographic range with Andean high-altitude origin and still inhabiting in their original range (Budnik and Brncic, 1974; Brncic, 1987). Nevertheless, interestingly, these two species coexist in nature where they exhibit similar life modes, food habits and reproductive sites (Godoy-Herrera and Connolly, 2007). Adult flies were collected in Til-Til (33°05′S, 70°55′W at 586 m above sea level). The climate at this locality is Mediterranean, with an annual mean precipitation of 376 mm, concentrated 65% in winter, from June to August. Precipitation is minimal from December to March, accounting for only 3% of the yearly total. Temperatures are highest from December to March (mean = 22°C), corresponding to austral summer, and lowest from June to August (mean = 7°C), during austral winter. Boher et al. (2010, 2012) describe the range limits of both species and populations at different acclimation temperatures. In the case of *D. melanogaster* upper lethal limit range from 36.7 to 37.8°C, and the lower lethal limit from −5.1 to −3.7°C. On the other hand, *D. gaucha* showed a upper lethal limit ranging from 35.9 to 37°C, and a lower lethal limit ranging from −11.3 to −5.5°C.

### Culture and experimental design

We used the fourth generation of field collected adults of *D. melanogaster* and *D. gaucha* to avoid potential environmental and maternal effects. Flies were reared in mass at 24°C in 250 ml glass vials with Burdick (1954) culture medium. At each generation, 40 adult flies were collected randomly from the rearing vials and transferred to fresh vials. After 3 days the adults were removed to prevent overlap between generations. Temperature range was set according to Boher et al. (2012) *Drosophila* thermal limits. Based on Bozinovic et al. (2011b) experimental design, during 15 days, adult flies were randomly assigned to four thermal treatments in climatic chambers; 17 ± 0°C (low mean, no variance = 17C), 17 ± 5°C (low mean, high variance = 17V), 24 ± 0°C (high mean, no variance = 24C), and 24 ± 5°C (high mean, high variance = 24V). The photoperiod was L:D = 12:12 h. 24°C was used as control temperature. We used hsp90, a molecular chaperone member of the heat shock protein family, which is upregulated in response not only to heat but also to cold stress (Colinet et al., 2010). After rearing flies at constant or fluctuating temperatures, we quantified *hsp90* mRNA expression in both species in each thermal scenario. Also hsp90 was the protein with more conserved alignment sequences in the primer design step. This is particularly relevant because *D. gaucha*, contrary to *D. melanogaster*, is not a model study so conserved alignments are critical to ensure an adequate primer performance.

RNA extraction was performed on 16-days old adults, after the 2 week acclimation period using Total RNA miniprep kit (Sigma). Each extraction was originated from a pool of 10 flies with three replicates per treatment. One microgram of total RNA was used in reverse transcription to cDNA, using the Transcriptor First Strand cDNA Synthesis Kit (Roche). Coding sequences of *hsp90* target gene and *rp49* housekeeping gene were retrieved from the GENBANK database. PCR primers were designed using Primer 3 module as follows: *hsp-90* forward 5′-CAAATCCCTGACCAACGACT-3′, *hsp-90* reverse 5′- TGATGTTGTTGCGCTTCTTC-3′; *rp49* forward 5′-CACCGGATTCAAGAAGTTCC-3′, *rp49* reverse 5′-GACGATCTCCTTGCGCTTCT-3′.

Real-time PCR were performed on a LightCycler 480 (Roche) system. PCR reactions were carried out using iQ SYBR Green Super Mix (Bio-Rad), and the crossing point (C_p_) were obtained. Samples were subjected to PCR amplification at 95°C for 5 min, 40 cycles at 95°C for 30 s, 54°C for 30 s, and 72°C for 30 s. A dissociation curve was carried out to ensure that there was only one product. A control without template was included in all batches. Amplification efficiency of each gene was validated by constructing a standard curve through four serial dilutions of cDNA. Data were analyzed following a method based in *C*_*p*_ according to Pfaffl (2001).

### Statistical analysis

Statistical analysis of gene expression values was carried out using the REST 2008 program (Relative Expression Software Tool V 2.0.7; Corbett Research; Pfaffl et al., 2002). This program calculates changes in gene expression between two groups, control and sample, using the corresponding distributions of *C*_*p*_-values as input. The program makes no assumptions about the distributions, evaluating the significance of the derived results by using the Pair-Wise Fixed Reallocation Randomization Test tool (Pfaffl et al., 2002).

## Results

**Figure 1:**
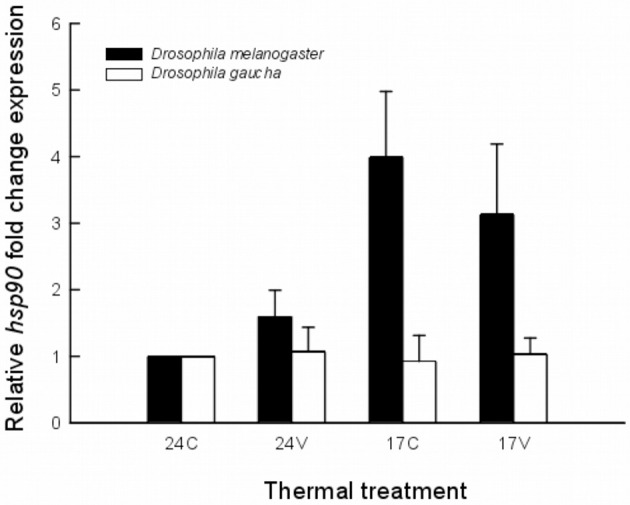
**Expression levels of *hsp90* mRNA in an invasive (*Drosophila melanogaster*) and a native (*Drosophila gaucha*) species acclimated to four thermal treatments**. Treatments were: 17 ± 0°C (low mean, no variance = 17C), 17 ± 5°C (low mean, high variance = 17V), 24 ± 0°C (high mean, no variance = 24C), and 24 ± 5°C (high mean, high variance = 24V). Results expressed as copies of hsp90 per copies of rp49. Data is representative of three biological replicates.

All qRT-PCR assays yielded specific products (i.e., single melting peak). The acclimation temperature of 24°C was used as control in *hsp90* expression analysis as was the rearing temperature for both species. The heat shock response varies greatly between the two species. *D. melanogaster* subtly increase *hsp90* mRNA expression when acclimated at 24V (Figure 1). The expression of *hsp90* mRNA was upregulated in *D. melanogaster* after acclimation at 17°C and also after acclimation at 17V without significant differences between them (Figure 1).

Statistical analysis using the REST 2008 program (Table 1) indicates that *hsp90* mRNA expression in the invasive *D. melanogaster* in both, low mean and low mean–low variance treatments, were significantly higher with respect to the other treatments and to the other species (fold change of 3.99; *P* = 0.027 and 3.13; *P* = 0.048, respectively). On the other hand, the native species, *D. gaucha* did not show *hsp90* mRNA overexpression at any of our thermal treatments (Table 1; Figure 1).

**Table 1:** **REST statistical analysis data of the expression values of *hsp90* and the range of standard errors (*SE*)**.

**Species**	**Thermal scenarios**	**Fold change**	**SE**	***P*-value**	**Result**
***D. melanogaster***					
	17 vs. 24C	3.991	3.558–4.544	0.027	UP
	17V vs. 24C	3.131	2.612–3.670	0.048	UP
	24V vs. 24C	1.602	1.429–1.819	0.056	–
***D. gaucha***					
	17 vs. 24C	0.924	0.746–1.136	0.622	–
	17V vs. 24C	1.035	0.928–1.162	0.751	–
	24V vs. 24C	1.067	0.890–1.260	0.465	–

*Data is representative of three biological replicates per treatment, each replicate originated from a pool of 10 flies. Data indicate that hsp90 is significantly upregulated (UP) only in individuals of the invasive species acclimated to constant and variable cold environmental conditions. Thermal treatments are: 17 ± 0°C (low mean, no variance = 17C), 17 ± 5°C (low mean, high variance = 17V), 24 ± 0°C (high mean, no variance = 24C), and 24 ± 5°C (high mean, high variance = 24V)*.

## Discussion

Adaptation to varying thermal environments depends on the temporal pattern of environmental changes and the physiological tolerance of each phenotype (Cavieres et al., 2016). In spite of the well-known role of climate change on biodiversity (Burroughs, 2007; Angilletta, 2009; Chown et al., 2010), the range of thermal conditions in time and space, its variability and how invasive and native animals respond to different climate change scenarios are still puzzling.

Bozinovic et al. (2013, 2016a,b) showed that those ectotherms that are continuously exposed to variations in environmental conditions deal with this variability through thermal acclimation and/or acclimatization, which impacts on the survival of natural populations. These authors also propose that if short time thermal variability changes in any of the directions forecast by climatologists, physiological approaches are necessary to predict the biodiversity consequences of climate change. In this vein, Colinet et al. (2015) showed that fluctuating ambient temperatures that remain within tolerant physiological ranges, usually improve performance in insects. Nonetheless, those which cover to extreme temperatures may have both positive impacts, allowing repair of damage accumulated to stressful conditions, or negative impacts from damage during successive exposures.

Biological invasions may interact with global warming, with invasions being favored with the increase in temperatures (Lejeusne et al., 2014; Barahona-Segovia et al., 2016). Zerebecki and Sorte (2011) proposed that invasive species should be less affected by global warming than native ones. For example, native and exotic shrimp respond differentially to increasing temperatures, —the exotic species having better performance at higher temperatures. This hypothesis however was tested for aquatic species, where temperature is less variable than in terrestrial ecosystems. Barahona-Segovia et al. (2016) observed that in native and invasive ladybugs the same hypothesis is not supported, because the native species is as eurythermic as the exotic one.

To predict responses to climate change, physiological ecologists must understand the patterns of thermal variation and the mechanisms by which animals cope with this variation (Burroughs, 2007; Dillon et al., 2010). Within this framework, we experimentally assessed the likely impact of three scenarios of climate change (Burroughs, 2007) on the heat shock response of invasive and native species. Interestingly, we observed that *hsp90* mRNA expression was indeed higher in the invasive species as has been reported in other studies (Henkel et al., 2009; Lockwood et al., 2010; Tomanek and Zuzow, 2010; Zerebecki and Sorte, 2011), but this species did not have a thermal range as wide as the native one (Boher et al., 2010), so we suggest that the biogeographic or evolutionary origin could be playing a role in the heat shock response of these species in different scenarios of climate change. In addition, we observed that the native species did not show *hsp90* mRNA overexpression as a result of our thermal treatments. It seems that, thermically variable as well as constant environments did not represent a stressful condition in species that evolved in harsh environments such as the Andes range. Although the heat shock response is ubiquitous, it varies among species and populations in several ways including the temperature at which HSP synthesis is induced (Somero, 1995).

Comparisons of the heat shock response in species evolutionary adapted to different temperatures, as in our study, have shown that the stress needed to induce HSPs is strongly related to the realized niche of the organism in question (Feder and Hofmann, 1999). For instance, among arctic fish HSPs are induced at around 5°C (Carpenter and Hofmann, 2000) and in thermophilic bacteria at nearly 100°C (Phipps et al., 1993). Among different species of *Drosophila*, it was shown that expression of *hsp70* is lower in lines frequently or continuously exposed to severe stress (Sorensen et al., 1999; Lansing et al., 2000). The interpretation was that the costs of HSPs expression related to fertility/fecundity, development and survival in populations frequently exposed to stress outweighed the benefits and that stress adaptation was achieved through some other means. The same pattern was subsequently found in natural populations of *Drosophila* (Sorensen and Loeschcke, 2001). According to these findings, the adaptive role of HSPs in connection to environmental stress resistance seems to occur during periods of relatively rare, unexpected extreme stress exposures and not during daily environmental fluctuations. On the other hand, the invasive species *D. melanogaster*, shows a marked heat shock response when faced to low mean and low mean-high variance treatments may be because those are stressful conditions from a species originated in tropical environments. Also *D. melanogaster* do present a subtle *hsp90* mRNA overexpression when acclimated at high mean-high variance treatments, again possibly because, although are temperatures within their natural range, variability is perceive as a stress condition.

In conclusion, although the invasive species has the ability to express HSPs over a wider range of thermal conditions than the native species, as have been seen in other invasive-native species comparisons (Henkel et al., 2009; Lockwood et al., 2010; Zerebecki and Sorte, 2011), we suggest that the heat shock response might be also associated with the thermal history of the species, more than a “invasive ecotype” *per-se* (Boher et al., 2012). Indeed, many reports support a lower scope for adaptive evolutionary responses to high temperatures, meaning a more conserved heat tolerance among ectotherms in general (Boher et al., 2010; Bozinovic et al., 2014). Thus, as with upper thermal limits of tolerances, our results suggest that historical biogeography may be an important feature associated with the biochemical response of species under current and future variable climatic scenarios.

## Author contributions

Conception and design: FB and FB. Acquisition and analysis: FB and NT. Drafting of the manuscript and revising it: FB, SE, and FB. All authors are approved the final version of the article.

## Funding

Partially funded by FONDECYT 3140424 to FB and FONDECYT BASAL FB 0002-2014 to FB and SE.

### Conflict of interest statement

The authors declare that the research was conducted in the absence of any commercial or financial relationships that could be construed as a potential conflict of interest.
